# Synergistic chemopreventive effects of curcumin and berberine on human breast cancer cells through induction of apoptosis and autophagic cell death

**DOI:** 10.1038/srep26064

**Published:** 2016-06-06

**Authors:** Kai Wang, Chao Zhang, Jiaolin Bao, Xuejing Jia, Yeer Liang, Xiaotong Wang, Meiwan Chen, Huanxing Su, Peng Li, Jian-Bo Wan, Chengwei He

**Affiliations:** 1 State Key Laboratory of Quality Research in Chinese Medicine, Institute of Chinese Medical Sciences, University of Macau, Macao 999078, China

## Abstract

Curcumin (CUR) and berberine (BBR) are renowned natural compounds that exhibit potent anticancer activities through distinct molecular mechanisms. However, the anticancer capacity of either CUR or BBR is limited. This prompted us to investigate the chemopreventive potential of co-treatment of CUR and BBR against breast cancers. The results showed that CUR and BBR in combination synergistically inhibited the growth of both MCF-7 and MDA-MB-231 breast cancer cells than the compounds used alone. Further study confirmed that synergistic anti-breast cancer activities of co-treatment of these two compounds was through inducing more apoptosis and autophagic cell death (ACD). The co-treatment-induced apoptosis was caspase-dependent and through activating ERK pathways. Our data also demonstrated that co-treatment of CUR and BBR strongly up-regulated phosphorylation of JNK and Beclin1, and decreased phosphorylated Bcl-2. Inhibition of JNK by SP600125 markedly decreased LC3-II and Beclin1, restored phosphorylated Bcl-2, and reduced the cytotoxicity induced by the two compounds in combination. These results strongly suggested that JNK/Bcl-2/Beclin1 pathway played a key role in the induction of ACD in breast cancer cells by co-treatment of CUR and BBR. This study provides an insight into the potential application of curcumin and berberine in combination for the chemoprevention and treatment of breast cancers.

Breast cancer, the leading cause of cancer death among females, has ranked the second among all new cancer cases in the world, and has been growing by 2.0% per year^1^. With the extensive application of surgery, radiotherapy, chemotherapy and endocrine therapy, the breast cancer mortality has been markedly reduced^2^. However, most anticancer drugs used for the treatment of breast cancer are the cytotoxic ones, which exhibits serious side effects on patients with breast cancer^3^. Besides, distinct complications occurred in patients with breast cancer after surgery or radiation, including cardiovascular diseases, axillary vein thrombosis and neuropathy and so on^4^. Meanwhile, chemotherapy was also found to possess little or no anticancer role in ER-positive breast cancer patients aged 40 years or less^5^. Although endocrine therapies specifically target estrogen and increase the survival rate of patients with breast cancer, drug-resistance is usually the main reason to limit the efficacy of breast cancer therapy^6^. Therefore, it is necessary for us to search for a novel approach for the prevention of breast cancers.

Cancer chemoprevention is described as a novel method to suppress or reverse the process of cancer using natural or synthetic compounds. Currently, the concept of chemoprevention has been expanded to target all stages of cancer development, including cancer initiation and progression^7^. Meanwhile, more and more researchers have exhibited increased interest in this field, since phytochemicals from dietary plants and herbs have emerged as a new source of the cancer chemoprevention and as an adjuvant of chemotherapy drugs^7^, which have the ability to prevent cancer initiation and progression through free-radical scavenging, DNA damage and apoptosis.

Apoptosis and autophagic cell death are the main forms of cell death, which play profound roles in cancer chemoprevention. Apoptosis eliminates aging cells and maintains homeostasis in organisms. Studies indicate that various types of cell stresses, including oxidative stress, ER stress and DNA damage, can trigger apoptosis^8^. Autophagy as a conserved pathway promotes cell survival by purging damaged organelles, glycogens and proteins^9^. However, autophagy may also induce cell death^10^. Recently, apoptosis and autophagy, as life and death partners, have been shown to affect each other by many complex mechanisms, including JNK/Beclin1/Bcl-2 pathways^11^.

CUR and BBR, respectively isolated from the root of *turmeric* and *Rhizoma coptidis*, are well-known phytochemicals for their multiple pharmacological properties, including anti-inflammation, neuroprotection, anti-diabetes, anti-cardiovascular diseases, anticancer, etc.^12^,^13^. CUR, a diarylheptanoid exhibits marked pro-oxidant activities^14^, which is connected with its chemical structure of the unsaturated carbonyl and phenolic hydroxyl groups^15^. ROS production induced by curcumin mediated apoptosis and autophagic death in cancer cells via modulating distinct cell survival and death pathways^16^. BBR, an isoquinoline alkaloid, could specifically bind with nucleic acids (DNA or RNA)^17^, and induce DNA damage in cancer cells through regulating the activity of DNA topoisomerase and ultimately leads to cell death^17^, which is associated with the DNA damage response (DDR). Consequently, DDR induces programmed cell death, such as apoptosis, when DNA damage cannot be successfully repaired^18^. DDR also often induces autophagic cell death in cancer cells^19^. A large body of research revealed that both CUR and BBR exhibited effective anticancer or chemopreventive activities with low toxicity in various cancer types through multiple mechanisms^20^,^21^. Notably, CUR and BBR have been applied in clinical trials for cancer chemoprevention of colorectal adenomas^22^,^23^. In addition, each of the compounds could enhance the sensitivity of cancer cells to conventional chemotherapeutic drugs^24^,^25^. Considering their distinct chemical properties, promising anticancer activities, multiple targets, low toxicity, and rich resources, it is intriguing to investigate whether combination of two renowned natural compounds CUR and BBR could exert synergistic chemopreventive effects, in particularly, on breast cancer cells.

In the present study, we demonstrated that co-treatment of CUR and BBR exhibited synergistic chemopreventive effects through inducing caspase-dependent apoptosis and autophagic cell death via activation of ERK and JNK/Beclin1/Bcl-2 signaling pathways, respectively, in MCF-7 and MDA-MB-231 breast cancer cell lines. Our results suggested that combination of curcumin and berberine could be a promising approach for breast cancer chemoprevention.

## Results

### Co-treatment of CUR and BBR exerts synergistic cytotoxicity against breast cancer cells

**Figure 1:**
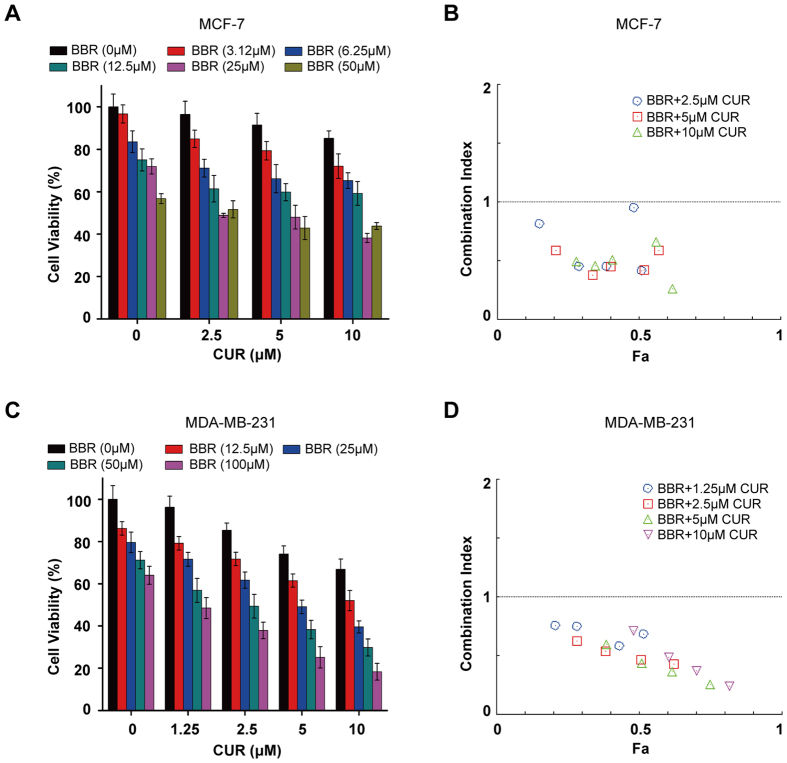
Co-treatment of CUR and BBR exerts synergistic cytotoxicity against breast cancer cells. MCF-7 (**A**) and MDA-MB-231 (**C**) cells were treated with indicated concentrations of CUR and BBR alone or in combination for 48 h. Cell viability was detected by MTT assay. The experiments were carried out in triplicate and each value represents means ± SD. Combination index for MCF-7 (**B**) and MDA-MB-231 (**D**) cells were analyzed by CompuSyn software. CI < 1, = 1, and >1 stand for synergistic, additive, and antagonistic effects, respectively. “Fa” refers to inhibitory rate. Values represent the mean ± SD (n = 3).

To determine the potential chemopreventive activity of CUR and BBR in combination, we firstly co-treated MCF-7 cells with increasing concentrations of CUR (2–10 μM) and BBR (3–50 μM) for 48 h. Cell viabilities were measured and quantified by MTT assay. The results showed that BBR treatment alone resulted in a dose-dependent growth inhibition in MCF-7 cell, with an IC_50_ value of 65.27 ± 4.67 μM (Fig. 1A). However, co-treatment of BBR with CUR of 2.5, 5.0, and 10 μM significantly enhanced the growth inhibition, as indicated by markedly decreased IC_50_ values of 34.85 ± 0.48, 22.51 ± 4.31, and 21.05 ± 1.25 μM, respectively. As shown in Fig. 1B, all CI values of these compounds in combination were less than one, suggesting that the growth inhibitory effect of these compounds in combination was synergistic rather than additive or antagonistic. BBR at 25 μM and CUR at 5 μM used alone caused slight cytotoxicity, however, combination treatment of them showed markedly synergistic effects (CI = 0.42). Therefore, these concentrations of CUR and BBR were used for latter experiments. Since the anticancer effects of conventional chemotherapy on triple-negative breast cancers is poor comparing to ER-positive breast cancers^26^, we tested the effect of CUR and BBR in combination on MDA-MB-231 cells, a triple-negative breast cancer cell lines. Similarly, BBR dose-dependently inhibited the growth of MDA-MB-231 cells with an IC_50_ value of 178.87 ± 3.16 μM, while the IC_50_ values of co-treatment of BBR with 2.5, 5.0, and 10 μM CUR decreased to 51.89 ± 4.57, 26.85 ± 2.77, and 14.85 ± 1.02 μM, respectively, indicating an enhanced cytotoxicity in the co-treatment groups (Fig. 1C). It is worth to note that all CI values of the tested combination treatments were less than 1 (Fig. 1D), further confirmed that the combination treatment also exhibited a synergistic cytotoxic effect (CI = 0.44) on MDA-MB-231 cells. These results showed that co-treatment of CUR and BBR synergistically killed both triple-negative MDA-MB-231 and ER-positive MCF-7 cells.

### Co-treatment of CUR and BBR induces apoptosis in breast cancer cells through activation of ERK signaling pathway

**Figure 2:**
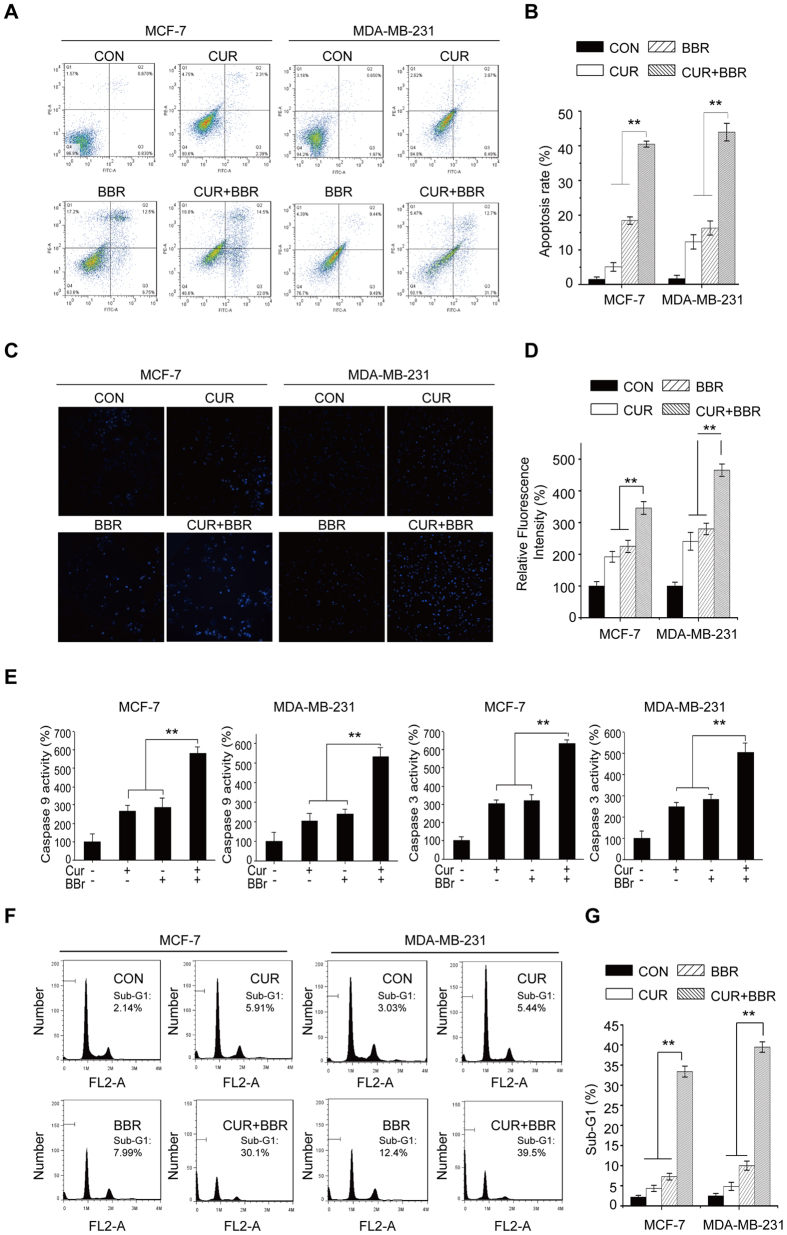
Co-treatment of CUR and BBR induces apoptosis in breast cancer cells. Cells treated with 5 μM CUR and 25 μM BBR alone and in combination for 48 h were analyzed for apoptosis using Annexia V-FITC/PI double staining assay (**A**), Hoechst 33342 staining and visualized by INCell Analyzer 2000 (20×) (**C**), caspase-3/9 detection using colorimetric assay kits (**E**), and Sub-G1 phase detection by flow cytometry (**F**). (**B**,**D**,**G**) were quantified results of (**A**,**C**,**F**) respectively. Values represent the mean ± SD (n = 3). **p < 0.01 compared with the co-treatment group.

**Figure 3:**
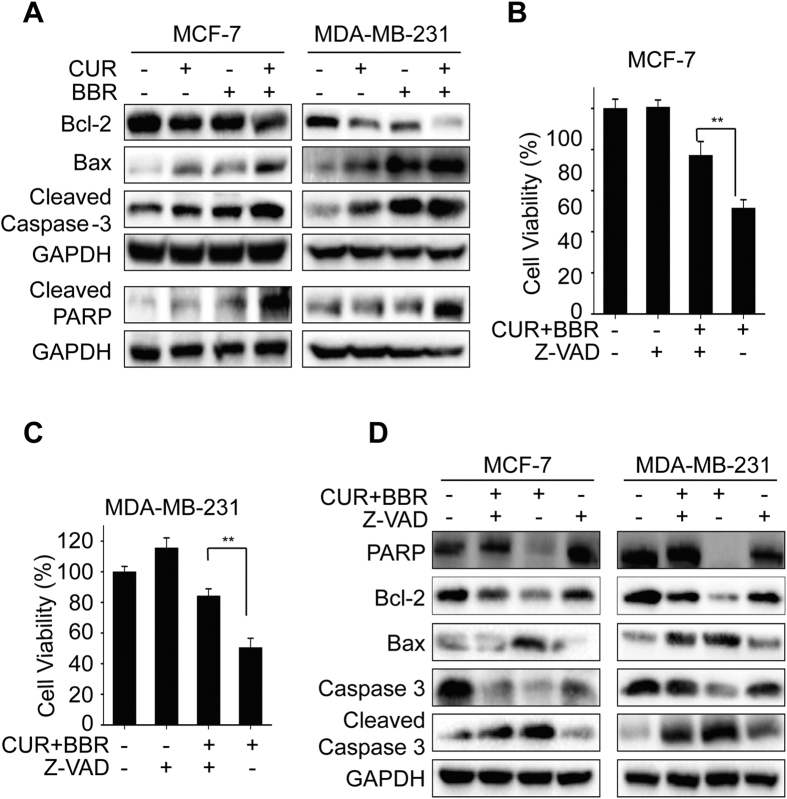
Apoptosis induced by co-treatment of CUR and BBR is via caspase-dependent pathway. Total proteins were extracted from cultured MCF-7 and MDA-MB-231 cells treated with 5 μM CUR and 25 μM BBR alone and in combination for 48 h. (**A**) The expression of Bcl-2, Bax, cleaved caspase-3, and cleaved PARP were analyzed by Western blotting as described in the “Materials and Methods”. MCF-7 and MDA-MB-231 cells pretreated with Z-VAD (10 μM) for 1 h and then treated with CUR and BBR in combination for 48 h for cell viability measurement by MTT assay (**B**,**C**) and determination of PARP, BAX, Bcl-2 and caspase-3 protein levels by Western blotting (**D**). GAPDH was used as the loading control. Values represent the mean ± SD (n = 3). **p < 0.01.

Next, we determined whether the synergistic cytotoxic effect of CUR and BBR is associated with apoptosis. Results from Annexin V/PI staining indicated that the apoptotic rates were higher in both MCF-7 and MDA-MB-231 cells co-treated with CUR and BBR (Fig. 2A,B) comparing to the groups of single treatment. In addition, there were more cells with brighter nuclei stained by Hoechst 33342 dye, inferring more apoptotic cells, in the co-treatment groups (Fig. 2C,D). Meanwhile, the activities of caspase-3 and -9 were higher in both cell lines co-treated with the two compounds comparing to that of either agent treatment alone (Fig. 2E). The accumulation of Sub-G1 phase is closely associated with the presence of apoptosis and is a marker of DNA damage. As shown in Fig. 2F,G, accumulation of sub-G1 cells induced by these compounds in combination was higher than the groups of either CUR or BBR treated alone. These data suggest that CUR and BBR in combination synergistically induces apoptosis in the two breast cancer cells. Our results revealed that the enhanced induction of apoptosis by combination treatment was further evidenced by decreased expression of Bcl-2 and increased expressions of Bax, cleaved caspase 3 and cleaved PARP (Fig. 3A). Interestingly, addition of the caspase inhibitor markedly suppressed the levels of Bax and cleaved caspase-3 expressions and increased the levels of total PARP, total caspase-3 and Bcl-2 expressions in the two breast cancer cell lines (Fig. 3D). Supporting this observation, the cell death induced by co-treatment was effectively suppressed by pan-caspase inhibitor (Z-VAD) in both MCF-7 and MDA-MB-231 cell lines (Fig. 3B,C). These results demonstrated that CUR and BBR in combination mainly induces apoptotic cell death in both ER-positive and triple-negative breast cancer cells in a caspase-dependent manner.

**Figure 4:**
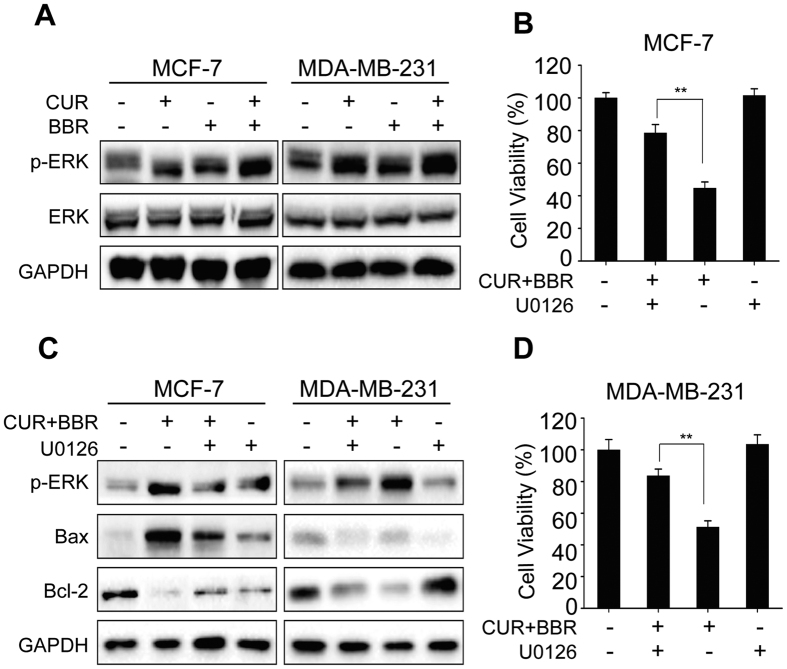
Apoptosis induced by co-treatment of CUR and BBR is mediated by activation of ERK. (**A**) Cells were treated with 5 μM CUR and 25 μM BBR alone and in combination for 48 h. Protein extraction and Western blotting were then performed to determine the levels of ERK and p-ERK. MCF-7 (**B**) and MDA-MB-231 (**C**) cells were pretreated with U0126 (10 μM) for 1 h and then treated with CUR and BBR in combination for 48 h. (**B**,**D**) Cell viability was measured using MTT assay. (**C**) Protein extraction and Western blotting were performed to determine the levels of p-ERK, Bax and Bcl-2. GAPDH was used as the loading control. Values represent the mean ± SD (n = 3). **p < 0.01.

Extracellular signal-regulated kinase (ERK), one of the members of MAPK (mitogen-activated protein kinase) family, is an important regulator of cell proliferation and survival^27^. However, it also could mediate cell death in some cell types^28^. Recent studies indicated that chemopreventive agents under certain conditions are able to induce cancer cell death via activation of ERK pathways^28^. In this study, we examined the role of ERK signaling pathways in apoptotic cell death induced by combination treatment of CUR and BBR. As shown in Fig. 4A, either CUR or BBR used alone increased the protein levels of phosphorylated ERK, while combination treatment of the two compounds caused more ERK phosphorylation. U0126, an ERK inhibitor, significantly suppressed the phosphorylation of ERK activated by the co-treatment (Fig. 4C). In addition, inhibition of ERK by U0126 also significantly reversed the increased Bax and decreased Bcl-2 expression levels and the cytotoxicity induced by the two compounds in combination in both cell lines (Fig. 4B–D). Collectively, these results suggested that co-treatment of CUR and BBR induced apoptotic cell death was mediated by up-regulated activation of ERK signaling pathway in both ER-positive and triple-negative breast cancer cells.

### Co-treatment of CUR and BBR induces autophagic cell death in breast cancer cells

**Figure 5:**
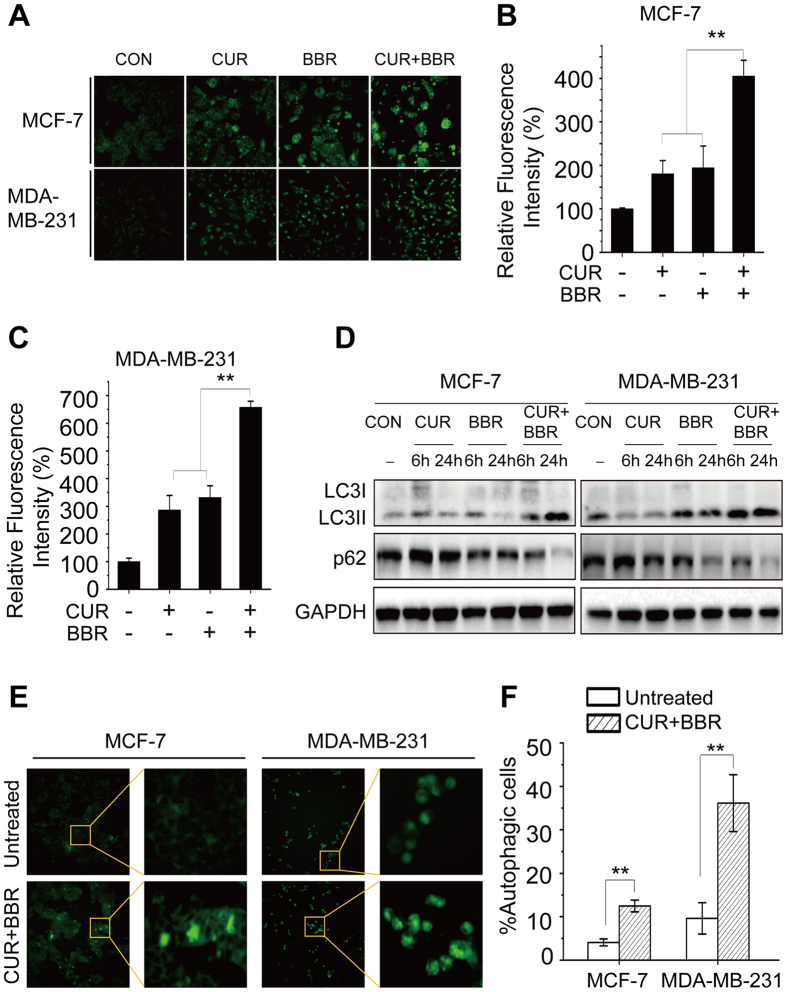
Co-treatment of CUR and BBR synergistically induces autophagy in breast cancer cells. MCF-7 and MDA-MB-231 cells were treated with 5 μM CUR and 25 μM BBR alone and in combination for 24 h. Autophagy was determined by MDC staining and observed using INCell Analyzer 2000 (20×) (**A**), detection of LC3-I, LC3-II and p62 protein levels using Western blotting (**D**), and detection of puncta morphology using EGFP-LC3 expressing plasmid transfection and observed under INCell Analyzer 2000 (**E**). (**B**,**C**) were quantified results of (**A**). (**F**) was quantified results of (**E**). Autophagic cells were quantified from random image fields totaling 200 cells. Values represent the mean ± SD (n = 3). **p < 0.01.

**Figure 6:**
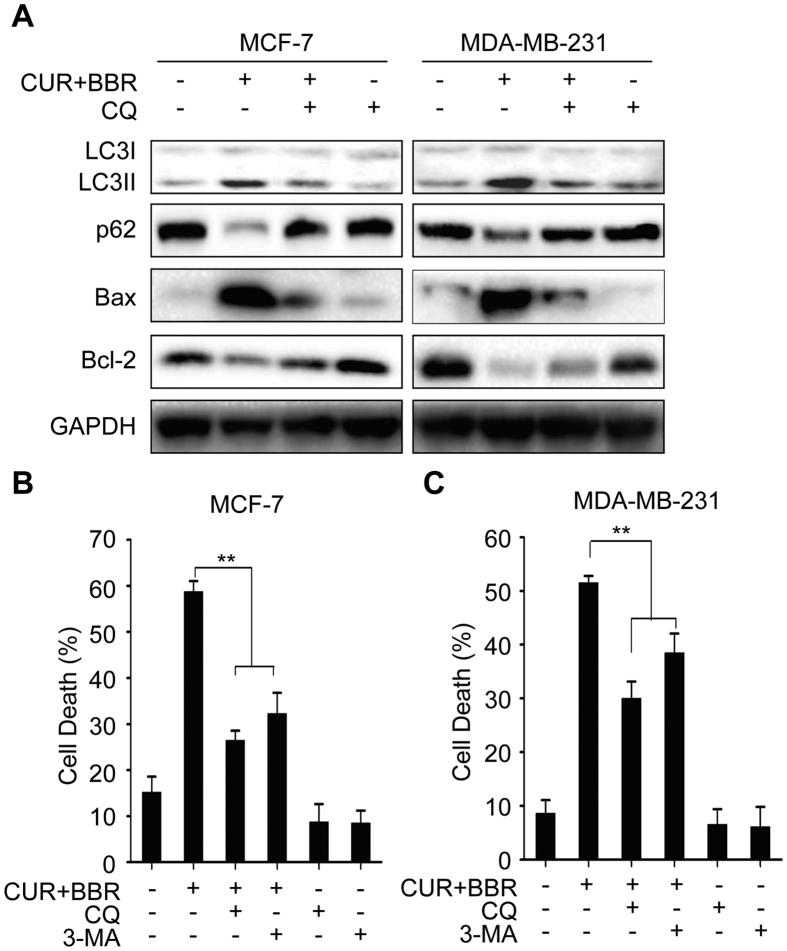
Autophagy contributes to the synergistic cytotoxicity of co-treatment of CUR and BBR. (**A**) MCF-7 and MDA-MB-231 cells were pretreated with CQ (10 μM) for 1 h and then co-treated with CUR (5 μM) and BBR (25 μM) for 24 h. Protein levels of LC3-I, LC3-II, p62, Bax and Bcl-2 were analyzed by Western blotting. The cells were co-treated with CUR (5 μM) and BBR (25 μM) with or without CQ (10 μM) or 3-MA (10 μM) pretreatment. Cell death were then measured using LDH assay in MCF-7 (**B**) and MDA-MB-231 (**C**) cells. Values represent the mean ± SD (n = 3). **p < 0.01.

In previous studies, it was found that CUR induced formation of autophagosome in human colon cancer cells^16^. BBR was also reported to induce autophagic death in many cell lines^29^. Here we investigate whether co-treatment of CUR and BBR enhances autophagy induction. The formation of autophagic vacuoles can be detected using MDC staining, which shows a punctate morphology in autophagic cells. As shown in Fig. 5A, both two control cell lines presented diffuse staining. BBR or CUR treatment alone resulted in the appearance of punctate structures. However, the number and size of punctate structures were markedly increased following co-treatment of these compounds comparing to either agent treatment alone in both breast cancer cell lines (Fig. 5A–C). Moreover, the potentiation of autophagy induction by the combination treatment was further evidenced by augmented accumulation of LC3-II and reduction of p62 (Fig. 5D), which are two key markers for the determination of autophagy. However, chloroquine (CQ) pretreatment markedly inhibited autophagy in the two breast cancer cells (Fig. 6A). We also monitored the turnover of LC3-II and its subcellular localization pattern using EGFP-LC3-expressing cells. The cells without treatment exhibited a diffuse localization of EGFP-LC3 (Fig. 5E), while prominent EGFP-LC3 puncta were observed in cells co-treated with curcumin and berberine (Fig. 5E). Quantification analysis indicated that there were 36.15% and 12.48% of EGFP-LC3 puncta expressing MDA-MB-231 and MCF-7 cells with co-treatment, respectively, compared with 9.61% and 4.07% of positive cells without any treatment (Fig. 5F). These results confirmed that CUR and BBR in combination synergistically stimulates the formation of autophagic flux in both ER-positive and triple-negative breast cancer cells.

To investigate the relationship between autophagy and cell death induced by CUR and BBR in combination, 3-MA and CQ were used to inhibit autophagy at early and late stages, respectively. We found that inhibiting autophagy with CQ and 3-MA greatly reduced cytotoxicity induced by these compounds in combination in both MCF-7 (Fig. 6B) and MDA-MB-231 cells (Fig. 6C). Meanwhile, inhibition of autophagy with CQ also markedly attenuated the increased Bax/Bcl-2 ratio in the two breast cancer cells (Fig. 6A). Collectively, these findings supported that CUR and BBR in combination may induce autophagic cell death in the two breast cancer cells.

### JNK/Bcl-2/Beclin1 signaling pathway plays a key role in autophagic cell death induced by co-treatment of CUR and BBR

**Figure 7:**
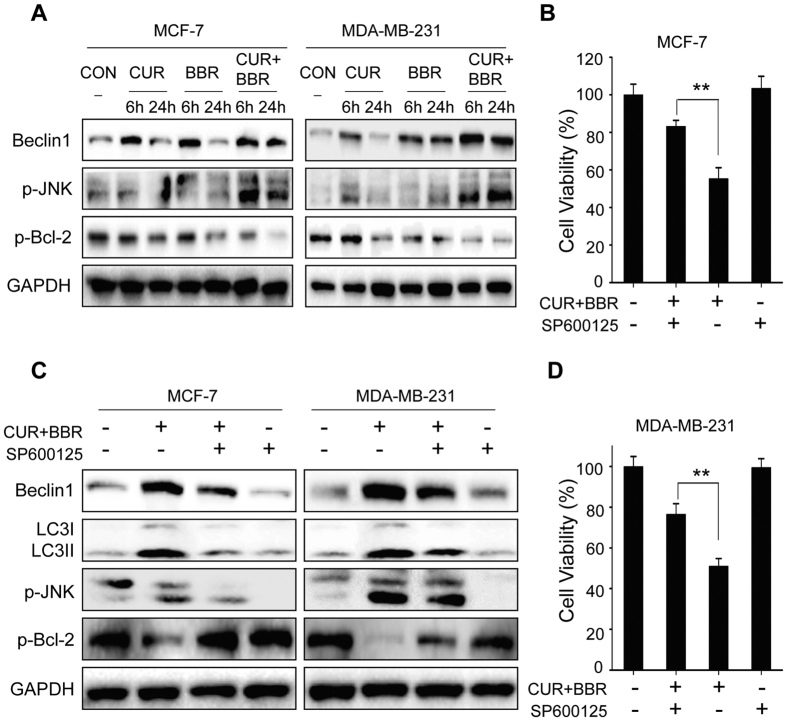
JNK/Bcl-2/Beclin1 signaling pathway plays a key role in autophagic cell death induced by co-treatment of CUR and BBR. (**A**) MCF-7 and MDA-MB-231 cells were treated with CUR (5 μM) and BBR (25 μM) alone and in combination for 6 h or 24 h. Protein extraction and Western blotting were performed to detect the levels of Beclin1, p-JNK and p-Bcl-2. GAPDH was used as the loading control. Cells co-treated with CUR (5 μM) and BBR (25 μM) with or without SP600125 (10 μM) pretreatment were subjected to measuring the levels of Beclin1, LC3-I, LC3-II, p-JNK and p-Bcl-2 by Western blotting (**C**), and determining the cell viability by MTT assay (**B**,**D**). Values represent the mean ± SD (n = 3). **p < 0.01.

Increasing evidence suggests that activation of JNK induces autophagy through phosphorylation of Bcl-2 and dissociation of Bcl-2/Beclin1 complex^11^. The phosphorylated Bcl-2 could be degraded by proteasome through ubiquitin-dependent mechanism under certain stresses^30^,^31^. Our results indicated that CUR and BBR treatment alone slightly increased the protein levels of p-JNK and Beclin1 and decreased p-Bcl-2 levels in the two breast cancer cell lines, while co-treatment of these two compounds remarkably enhanced the regulatory effects on these proteins (Fig. 7A). However, co-treatment with JNK inhibitor SP600125 significantly attenuated the decreased p-Bcl-2 and increased Beclin1 as well as LC3-II induced by combination treatment with the two compounds (Fig. 7C). In addition, inhibition of JNK by SP600125 markedly reduced the cytotoxicity induced by these two compounds in combination (Fig. 7B,D). These results suggest that CUR and BBR in combination induces autophagic cell death through JNK-mediated phosphorylations, phosphorylated Bcl-2 and dissociation of Bcl-2/Beclin1 complex in both breast cancer cell lines.

## Discussion

In this study, we tested synergistic chemopreventive effects of CUR and BBR in combination on ER-positive and triple-negative breast cancer cell lines. The data convincingly demonstrated that co-treatment of CUR and BBR markedly exerted a synergistic growth inhibitory effect on both cell lines through triggering apoptosis and autophagic cell death. The apoptosis involved ERK activation and caspase-dependent pathway, while the autophagic cell death involved JNK activation, Bcl-2 phosphorylation and the dissociation of Beclin1/Bcl-2 complex. Taken together, co-treatment of CUR and BBR markedly inhibits cell growth and might be a promising strategy for breast cancer chemoprevention.

CUR and BBR are renowned phytochemicals which possess distinct chemical structures and exhibit remarkable anticancer activities via different molecular mechanisms. However, their anticancer capability is compromised due to the limited intrinsic cytotoxic potential and nonspecific cytotoxicity when high dosage is applied. We speculate that combination treatment of CUR and BBR could target different molecules or pathways simultaneously and therefore exert synergistic cancer chemopreventive effects. In our experiments, CUR (2–10 μM) and BBR (3–50 μM) treatment alone resulted in a dose-dependent reduction of cell growth, accounting for 3–15% and 3–43% reduction in MCF-7 cells, respectively. Similar results were found in MDA-MB-231 cells, accounting for 3–34% and 13–36% reduction, respectively. These results were consistent with previous reports^32^,^33^. However, CUR and BBR co-treatment at the same range of concentrations caused growth inhibition of 15–62% and 20–82% in MCF-7 and MDA-MB-231 cells, respectively, which were significantly higher than the compounds treated alone. Besides, all CI values of these compounds in combination were less than one, indicating that these two compounds in combination markedly exerted synergistic growth inhibitory effects on MCF-7 and MDA-MB-231 cells.

Apoptosis, the major type of programmed cell death (PCD), is an active cell suicide process. It can be divided into two categories: caspsase-dependent and caspase-independent^9^. Among them, apoptosis in most cases is mediated in a caspase-dependent manner through death receptor (extrinsic) or mitochondrial (intrinsic) pathways. Mitochondria plays crucial roles in the intrinsic apoptosis. Bcl-2 family proteins, including anti-apoptotic Bcl-2 and pro-apoptotic Bax proteins, which mainly regulate mitochondrial membrane potential accompanied by the release of mitochondrial proteins, such as cytochrome c^34^. The release of cytochrome c from mitochondria to cytosol activates caspase-9, the initiator of intrinsic pathway that binds to Apaf-1 and cytochrome c and then activates caspase-3 and subsequently leads to apoptosis. In this study, we found that CUR at 5 μM and BBR at 25 μM treatment alone slightly induced apoptosis (<20%) in both breast cancer cell lines, however, combination treatment synergistically augmented apoptosis induction to more than 40% in MCF-7 and MDA-MB-231 cells (Fig. 2A,B). Meanwhile, the activities of caspase-3 and -9 and accumulation of Sub-G1 phase were also markedly increased in both cell lines co-treated with these two compounds comparing to that of either agent alone (Fig. 2E–G). In addition, we observed that the co-treatment more greatly decreased the expression of Bcl-2 and increased expressions of Bax, cleaved caspase-3 and cleaved PARP comparing to either agent treated alone. Addition of the pan-caspase inhibitor Z-VAD, however, prominently diminished the effects of CUR and BBR co-treatment on the protein levels (Fig. 3D) and cell viability of both MCF-7 cells and MDA-MB-231 cells. These data suggested that the synergistic pro-apoptotic activities of the co-treatment might be through inducing the caspase-dependent mitochondrial pathway^35^, which is in consistent with previous reports when either compound used alone^36^,^37^.

Interestingly we found that combination treatment of CUR and BBR caused more ERK phosphorylation compared with groups of CUR and BBR treated alone in both cell lines. Pretreatment with ERK inhibitor markedly restrained phosphorylation of ERK, Bax/Bcl-2 ratio and the cytotoxicity induced by these two compounds. These results indicated that co-treatment of CUR and BBR induced apoptosis in the breast cancer cells via activation of ERK pathway. ERK plays complicated roles in cell proliferation and apoptosis. On the one hand, ERK activation has generally been found to prevent apoptotic cell death and induce cell proliferation through blockage of a critical component of the cell-intrinsic death machinery and enhancement of the expression of pro-survival genes through the Ras-Raf-MEK-ERK signaling pathway^27^. On the other hand, a number of studies indicated that activation of ERK could trigger apoptotic cell death depending upon the cell types and stimuli^28^. It was suggested that the intensity and duration of pro- versus anti-apoptotic signals transduced by ERK determined the cell fate^38^. ERK activation by sustained DNA damage could lead to p53 activation and subsequently induce both growth arrest and apoptosis. In addition, ERK-mediated cell apoptosis may be associated with suppression of nuclear translocation and cytosolic retention of activated ERK through its interaction with the pro-apoptotic proteins Bik, PEA-15 and DAPK, and activation of these protein scaffolding^38^. The detailed mechanisms remain to be further investigated.

Autophagy is an evolutionarily conserved catabolic process which is primarily used for cellular adaptive response to deficiency in nutrient resources. Autophagy can also be broadly induced by multiple external stimuli such as ionizing radiation and cytotoxic chemicals, including anticancer reagents. It plays dual roles in cancers, i.e. pro-survival or pro-death of cancer cells, depending on types of cells and stimuli, and stimulus concentration and duration of treatment^39^,^40^. Although autophagy is generally regarded as a cytoprotective process, prolonged stress and sustained autophagy could eventually lead to cell death^41^. Autophagic cell death (ACD) has been proposed as an additional mechanism for cell death in the absence of the apoptosis signaling pathways^42^. Here we found that CUR and BBR could significantly induce autophagy in both MCF-7 and MDA-MB-231 cells, which is in accordance with previous reports^43^,^44^. Interestingly, co-treatment of these two compounds much more potently induced autophagy in the breast cancer cells, as evidenced by the increased number and size of punctate structures in cells (Fig. 5), which are the feature of autophagic vacuole formation, higher levels of LC3-II turnover and p62 protein degradation, when compared to either agent treated alone. The turnover of LC3-II and its subcellular localization pattern were also observed using EGFP-LC3-expressing cells. Quantification analysis indicated that there were 36.15% and 12.48% of EGFP-LC3 puncta expressing in MDA-MB-231 and MCF-7 cells co-treated with CUR and BBR, respectively, comparing to 9.61% and 4.07% of positive cells without treatment. The differential responses to the autophagy induction by the co-treatment might be due to different background of the two breast cancer cell lines. Similar phenomenon was observed that MDA-MB-231 cells were more sensitive to rapamycin-induced autophagy than MCF-7 cells^45^. The presumed reason is that autophagy could be mediated by AKT/mTOR and/or JNK/Bcl-2/Beclin1 pathways, which are cell type-dependent^46^. Inhibition of autophagy by CQ and 3-MA, which were used to suppress autophagy at early and late stages, respectively, greatly reduced the cytotoxicity induced by combination treatment in both cell lines. Furthermore, inhibition of autophagy by CQ also markedly attenuated the increased Bax/Bcl-2 and LC3II/LC3I ratios and the decreased p62 expression. These results convincingly demonstrated that the enhanced cytotoxicity by co-treatment of CUR and BBR could be also mediated, at least partially, by inducing autophagic cell death in both ER-positive and ER-negative breast cancer cells.

In mammalian cells, anti-apoptosis protein Bcl-2 binds to autophagy protein Beclin1 and blocks the initiation of autophagosome formation in normal conditions^47^. JNK signaling pathway has been proved to modulate autophagy in response to cell stresses^47^. Increasing evidence shows that JNK activation facilitates the phosphorylation of Bcl-2 and dissociation of the Beclin1/Bcl-2 complex, and the dissociated active Beclin1 induces autophagy^47^. Hence, Bcl-2 regulates both apoptosis and autophagy, and Beclin1/Bcl-2 complex functions as a toggle switch in triggering autophagy^48^. In this study, our results demonstrated that co-treatment of CUR and BBR more strongly increased the protein levels of phosphorylated JNK and Beclin1, and decreased the phosphorylated Bcl-2, suggesting that the co-treatment activated JNK signaling pathway, leading to phosphorylation of Bcl-2 and dissociation of Beclin1, and consequently resulted in autophagic cell death in the two breast cancer cells. Inhibition of JNK by SP600125 significantly increased p-Bcl-2 and decreased Beclin1 levels, and reduced the cytotoxicity of the co-treatment of CUR and BBR, further confirm our speculation. Numerous studies have suggested that phosphorylation of Bcl-2 could strengthen the anti-apoptotic activity of Bcl-2 and could inhibit autophagy^49^. Others reported that phosphorylation of Bcl-2 may lead to its ubiquitination and proteasomal degradation and disarm its anti-apoptotic function^30^,^31^. In the current study, we found that phosphorylated Bcl-2 induced by JNK activation was significantly reduced following the combination treatment of CUR and BBR. In contrast, inhibition of JNK markedly restored Bcl-2 phosphorylation. JNK can be activated by multiple stimuli or pathways, including oxidative stress or genotoxic agents, growth factors, inflammatory cytokines, and ligands of G-protein-coupled receptors (GPCRs), among which, the stresses or genotoxic agents are the most powerful inducers of JNK^50^. We suppose that activation of JNK by co-treatment of CUR and BBR is through oxidative stress response and/or genotoxic pathways since CUR is a potent pro-oxidant^14^ and BBR could insert DNA and interrupt DNA replication^17^. NF-κB signaling system plays an important role in the regulation of autophagy^51^. It could inhibit autophagy through increasing the expression of autophagy repressors A20, Bcl-2, and NLRP receptors, and suppressing autophagy inducers BNIP3, JNK and ROS. Both CUR and BBR are known to be NF-κB inhibitors^52^,^53^, which could explain, at least partially, the synergistic induction of autophagy in the breast cancer cells by the combination treatment.

**Figure 8:**
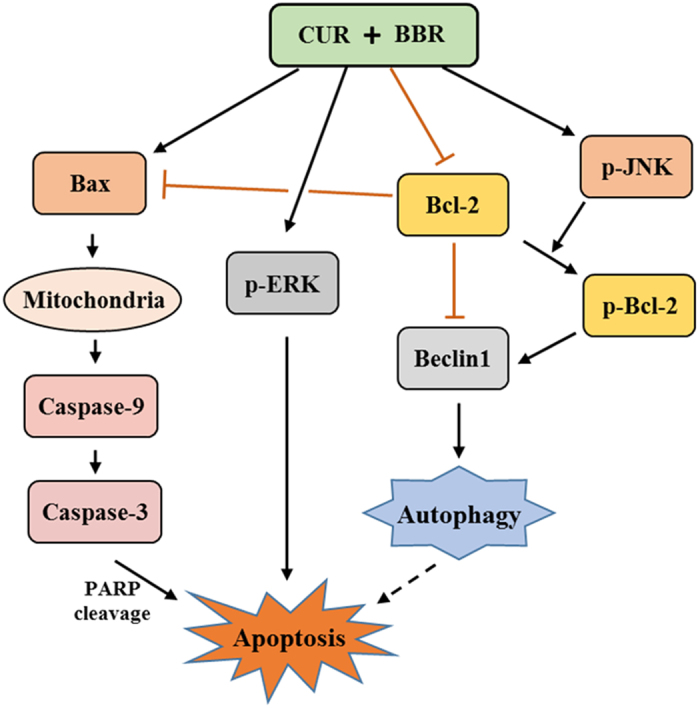
Schematic diagram showing the proposed mechanisms for the synergistic chemopreventive effects of CUR and BBR on human breast cancer cells. CUR and BBR in combination induces caspase-dependent apoptosis in breast cancer cells through ERK activation. Meanwhile, combination of these two compounds causes JNK activation, phosphorylation of Bcl-2, and dissociation of the Beclin1/Bcl-2 complex, leading to autophagic cell death in breast cancer cells.

In conclusion, the present study demonstrated that co-treatment of CUR and BBR exhibited synergistic anticancer effects on both ER-positive and ER-negative breast cancer MCF-7 and MDA-MB-231 cells via induction of apoptotic and autophagic cell death. The co-treatment-induced apoptosis was associated with the activation of ERK signaling pathway, and the autophagic cell death was likely attributable to the activation of JNK, followed by phosphorylation of Bcl-2 and dissociation of Beclin1/Bcl-2 complex (Fig. 8). This study strongly suggests that curcumin and berberine in combination could be used for the prevention and treatment of breast cancers.

## Materials and Methods

### Chemicals and reagents

Curcumin and berberine with the purity greater than 99%, thiazolyl blue tetrazolium bromide (MTT), Hoechst 33342, dimethyl sulfoxide (DMSO), chloroquine diphosphate salt (CQ), 3-methyladenine (3-MA), and monodansylcadaverine (MDC) were obtained from Sigma Chemical Co (St. Louis, MO, USA). Z-VAD, caspase-3 and caspase-9 activity assay kits, LDH assay kit, Annexin V-FITC/ propidium iodide (PI) apoptosis detection kit, cell cycle and apoptosis analysis kit, U0126 (ERK1/2 inhibitor), and SP600125 (JNK inhibitor) were purchased from Beyotime Institute of Biotechnology (Nanjing, Jiangsu, China). Dulbecco’s Modified Eagle Medium (DMEM) and FBS were purchased from Gibco (Carlsbad, CA, USA). Polyclonal antibodies against Bax, Bcl-2, cleaved caspase-3, PARP1 and Cleaved PARP1, SQSTM1/p62, LC3-II, and Beclin1 were purchased from Proteintech (Chicago, IL, USA). Monoclonal antibodies against caspase-3, GAPDH, ERK1/2, Phospho-ERK1/2 (Thr202/Tyr204), JNK, Phospho-JNK (Thr183/Tyr185), Bcl-2, and Phospho-Bcl-2 (Ser70) were obtained from Cell Signaling Technology (Danvers, MA, USA). HRP-labeled goat anti-rabbit IgG (H + L) was supplied by Beyotime Institute of Biotechnology (Nanjing, Jiangsu, China). The plasmid pEGFP-LC3 and Lipofectamine^TM^ 2000 were purchased from Addgene (Cambridge, MA, USA) and Invitrogen (Carlsbad, CA, USA), respectively.

### Cell culture and drug treatments

Human breast MCF-7 and MDA-MB-231 cells were purchased from the American Type Culture Collection (Rockville, MD, USA). Cells were maintained in DMEM medium containing 10% (v/v) fetal bovine serum and 1% (v/v) penicillin/streptomycin, and incubated at 37 °C in a 5% CO_2_ incubator (Thermo, MA, USA). For the following *in vitro* experiments, CUR (20 mM), BBR (50 mM), Z-VAD (10 mM), CQ (10 mM), 3-MA (10 mM), U0126 (10 mM) and SP600125 (10 mM) powders were dissolved in DMSO as stock solutions and then diluted with fresh medium containing 10% FBS. Cells were pretreated with Z-VAD (10 μM), CQ (10 μM), 3-MA (10 μM), U0126 (10 μM) and SP600125 (10 μM) for 1 h before co-treatment of CUR and BBR. Moreover, the final concentration of DMSO in both MCF-7 and MDA-MB-231 cells treated with different concentrations of compounds was less than 0.1%.

### Cell viability and combination index analysis

Cell viability was measured using MTT method. Briefly, cells were seeded in 96-well plates at a density of 5000 cells per well in 100 μl medium. After overnight incubation, increasing concentrations of CUR or BBR were added to the wells and cultured for 48 h. Then, 10 μl of MTT (0.50 mg/ml in PBS) was added to the wells and incubated for 4 h at 37 °C.The mixture was removed and 100 μl DMSO was added to the wells. The OD values of the wells were detected at 570 nm by SpectraMax M5 microplate reader (Molecular Devices, USA). For drug combination experiments, cells were co-treated with CUR and BBR for 48 h. The data were analyzed by CompuSyn software with the results showed in combination index (CI) values, where CI value <1, =1, and >1 refer to synergetic, additive, and antagonistic effects, respectively^54^.

### LDH assay

LDH assay was used to determine the cytotoxic effect of CUR and BBR in combination on MDA-MB-231 and MCF-7 cells with or without CQ or 3-MA pretreatment according to the manufacturer’s instruction (Beyotime, Jiangsu, China).

### Annexin V-FITC/PI double staining

Flow cytometry (FCM) was carried out to evaluate apoptosis in MDA-MB-231 and MCF-7 cells with Annexin V-FITC/PI double staining. Briefly, cells were collected and washed with cold PBS, stained with Annexin V-FITC and PI, and incubated in the dark for 15 min. Then, the cells were detected by FACS Canto^TM^ (BD, CA, USA). The distributions of apoptotic cells were calculated using FlowJo software version 7.6.1 (Ashland, OR, USA).

### Hoechst 33342 staining

Cells were fixed with 4% paraformaldehyde for 20 min and washed three times with cold PBS. Then, these cells were incubated with Hoechst 33342 (1 mg/ml), which was diluted 1000 times with PBS. The fluorescent morphology changes of cells were determined by INCell Analyzer 2000 (GE Healthcare, USA). Cells with the characteristics of nuclear condensation, apoptotic body, and brighter blue fluorescence in the nucleus were identified as apoptotic cells.

### Sub-G1 DNA content analysis

For Sub-G1 DNA content analysis, cells were washed with cold PBS, fixed with ice-cold 70% ethanol and placed at −20 °C for 24 h, and then stained with PI for 15 min. Cells were detected by FACS Canto^TM^ (BD, CA, USA). The distributions of cell cycle were calculated using FlowJo software version 7.6.1. The ratios of apoptotic cells are expressed as the percentage of Sub-G1 DNA content.

### Caspase 3/9 activity assay

caspase 3 and 9 activities were carried out using caspase-3 and -9 activity assay kits (Beyotime, Jiangsu, China) according to the manufacturer’s instructions. Briefly, cells were washed with cold PBS, lysed by adding 100 μl of lysis buffer to the wells, incubated for 15 min at 4 °C and centrifuged for 15 min at 17500 g. Then, 10 μl of supernatants, 10 μl of Ac-DEVD-pNA/ Ac-LEHD-pNA (2 mM) and 80 μl of detecting buffer were mixed and incubated with the cells at 37 °C for 120 min. Cells without treatment of Ac-DEVD-pNA/ Ac-LEHD-pNA were used as the blank control. The OD value was detected at 405 nm. Caspase-3/9 activity was calculated using the following formula: caspase-3/9 activity (%) = (OD_A405,sample_ − OD_A405,blank_)/OD_A405,sample_ ×100.

### MDC staining

Cells were washed with PBS and incubated with MDC (20 μM) in PBS at 37 °C for 20 min. After the incubation, cells were fixed with 4% paraformaldehyde for 20 min and examined by INCell Analyzer 2000 (GE Healthcare, USA). For fluorescence analysis, the fluorescence intensity of MDC staining dots indicative of autophagy in cells without treatment was normalized to that of control sample. The level of control sample was set to 100% and the relative level of MDC staining dots in cells treated with CUR or BBR alone and in combination was calculated according to the following formula: Relative fluorescence intensity (RFI) % = T/C ×100, where T and C stand for the fluorescence intensity of the treatment group and control group, respectively.

### Transient transfection

Cells were washed two times with Opti-MEM medium that does not contain antibiotics and serum. Then, the cells were transfected with plasmid expressing pEGFP-LC3 using Lipofectamine^TM^ 2000 (Invitrogen, USA) for 12 h. The medium was replaced by the complete medium containing 10% FBS and antibiotics and cells were treated with CUR (5 μM) and BBR (25 μM) for 24 h and examined by INCell Analyzer 2000 (GE Healthcare, USA).

### Western blotting

The collected cells were lysed with the ice-cold RIPA buffer containing 1% phenylmethylsulfonyl fluoride (PMSF) and 1% protease inhibitor cocktail (Thermo, Rockford, IL) for 20 min and centrifuged at 12500 rpm for 15 min at 4 °C. Total protein concentrations of the lysates were assessed using BCA protein quantitation kit (Thermo, Rockford, IL). Protein samples (25 μg) were separated by SDS-polyacrylamide gel electrophoresis in 5–12% gels and then transferred onto methanol activated PVDF membranes at 20 V for 1.5 h. The PVDF membranes were blocked with 5% skim milk for 1 h, probed with the indicated primary antibody and blotted with the secondary anti-rabbit antibody for 1 h at room temperature. Protein bands were detected by Bio-Rad ChemiDoc^TM^ (Hercules, CA, USA).

### Statistical Analysis

All the statistical analyses were carried out by SPSS version 13.0 for Windows. All the data were expressed as means ± SD of three independent experiments. One-way ANOVA and LSD’s Post Hoc Test were used to analyze all experimental data. Statistical significance was accepted at the level of *p* < 0.05.

## Additional Information

**How to cite this article**: Wang, K. *et al.* Synergistic chemopreventive effects of curcumin and berberine on human breast cancer cells through induction of apoptosis and autophagic cell death. *Sci. Rep.* **6**, 26064; doi: 10.1038/srep26064 (2016).
